# Plasma Citrulline in the Diagnosis and Follow-Up of Celiac Disease

**DOI:** 10.3390/children12010041

**Published:** 2024-12-30

**Authors:** Alicia Isabel Pascual Pérez, Elene Larrea Tamayo, Santiago Jiménez Treviño, David González Jiménez, David Pérez Solís, Cristina Molinos Norniella, Juan José Díaz Martín

**Affiliations:** 1 Pumarín Primary Care Center, 33011 Oviedo, Spain; 2 Iztieta Primary Care Center, 20100 Errenteria, Spain; 3 Central University Hospital of Asturias, 33011 Oviedo, Spain; 4 San Agustín University Hospital, 33401 Avilés, Spain; 5 Cabueñes University Hospital, 33394 Gijón, Spain

**Keywords:** citrulline, celiac disease, amino acid, intestinal absorption

## Abstract

Background/Objectives: Citrulline, an amino acid produced by small bowel enterocytes, has been proposed as a potential marker of intestinal absorptive capacity. The aim of this study is to evaluate whether measuring citrulline levels could be useful for celiac disease (CD) patients, both at the time of diagnosis and during follow-up. Methods: A multicenter prospective study was conducted. Citrulline levels were measured and compared in 93 pediatric patients classified into three groups. Group A: 28 patients with newly diagnosed CD. In this group, an additional measurement was performed after 3–6 months on a gluten-free diet (GFD). Group B: 32 patients with a prior CD diagnosis and on a GFD for at least 6 months. Group C: 33 healthy controls. Citrulline levels were correlated with clinical and laboratory variables, including serological markers. Statistical analysis: t-tests for paired groups and independent groups, Pearson and Spearman correlation tests. Results: Newly diagnosed CD patients had lower citrulline levels compared to those on a GFD for more than 6 months (27.13 vs. 32.42 µmol/L; *p* > 0.05). Citrulline levels were nearly identical between healthy controls and CD patients on a GFD for more than 6 months (32.48 vs. 32.42 µmol/L; *p* > 0.05). Starting a GFD led to a significant increase in citrulline levels in group A (from 27.13 to 37.43 µmol/L, *p* < 0.001). Conclusions: Plasma citrulline could serve as a valuable marker for mucosal recovery in the follow-up of diagnosed celiac patients adhering to a GFD.

## 1. Introduction

Celiac disease (CD) is a systemic immune-mediated disorder triggered by the consumption of gluten and related prolamins in genetically predisposed individuals [1].

Its etiology is multifactorial, involving genetic factors (predominantly HLA DQ2 and DQ8), environmental factors (gluten exposure), and immunological components. It involves a permanent intolerance to dietary gluten proteins, present in wheat (gliadins and glutenins), rye (secalins), and barley (hordeins). The main pathophysiological consequence is patchy atrophy of the intestinal mucosa, leading to a subsequent reduction in its absorptive surface area [2].

The English physician Samuel Gee was the first to describe the classic clinical presentation of this disease [3]. However, it is now known that its presentation can be highly variable, with non-classical or asymptomatic forms becoming increasingly common [4].

Diagnosis has traditionally relied on a combination of suggestive symptoms and signs, detection of elevated serological markers, genetic testing, and compatible histology.

The initial recommended screening test for CD in these patients is, alongside total serum IgA quantification, the measurement of tissue transglutaminase antibodies (tTG-IgA) confirmed in a second sample with endomysial antibodies. The gold standard for diagnosis is duodenal biopsy [5], with lesions classified as Marsh type 2 or 3 considered consistent with CD [6]. However, villous atrophy is not specific to CD.

In 2012, for the first time, the possibility of diagnosing CD without biopsy was proposed for specific patients presenting with compatible symptoms, serology levels 10 times above the upper limit of normal, and confirmed genetic susceptibility [1]. The latest guidelines from the European Society for Paediatric Gastroenterology, Hepatology, and Nutrition (ESPGHAN) [7], in the pursuit of less invasive diagnostic methods and supported by studies validating the reliability of serological testing [8], advocate for this approach.

The only effective treatment remains a lifelong gluten-free diet (GFD) [9]. Follow-up is based on monitoring clinical progress (symptoms and growth) and serological markers [10,11,12].

Citrulline is a non-essential amino acid synthesized in the enterocytes of the small intestine (duodenum and jejunum) from glutamine and other amino acid derivatives. It is not incorporated into protein structures and circulates freely in plasma. It is an intermediate in the urea cycle. In mammals, it is used for arginine synthesis in various cells (mainly the kidneys), which in turn serves as a precursor for nitric oxide. Thus, it plays a fundamental role in maintaining homeostasis [13].

Citrulline has been proposed as a potential marker of intestinal function because it positively correlates with the absorptive intestinal mass. Additionally, it has been used as a predictive marker for weaning off parenteral nutrition in patients with short bowel syndrome [14]. In untreated celiac patients, intestinal mucosal atrophy is observed, resulting in a reduced absorptive surface. Upon gluten withdrawal from the diet, progressive recovery of the mucosa occurs until complete normalization. Despite advances in CD diagnostics, no single test is entirely definitive. The advantage of citrulline compared to current diagnostic tests lies in its non-invasive nature, relatively low cost, objective results, and its potential to provide information about the state of the mucosa. This could complement the diagnosis and follow-up of these patients.

The primary aim of the study was to assess the utility of plasma citrulline levels in children with suspected CD at the time of diagnosis, and as a marker of mucosal recovery and adherence to treatment following the initiation of a GFD.

## 2. Materials and Methods

This was a multicenter prospective study with two phases: cross-sectional and longitudinal. The study was conducted at a regional referral hospital and two local hospitals.

Patients were classified into three groups. The initial sample size in the study design was 30 patients per group. Group A: Patients with newly diagnosed CD before starting a GFD. Diagnosis was conducted following the 2012 ESPGHAN guidelines [1]. Patients were included without biopsy if they presented suggestive symptoms, positive serology (IgA anti-tissue transglutaminase antibodies (tTG-IgA) exceeding 10 times the upper limit of normal, confirmed by anti-endomysial antibodies in a second sample), and demonstrated genetic susceptibility (positive HLA-DQ2 or DQ8). For other cases, histological findings consistent with CD were also required. Group B: Patients with confirmed CD who had been on a GFD for at least six months. Group C: Healthy controls. These were patients undergoing blood tests for routine hospital visits or scheduled surgical procedures. Children with intercurrent infections, metabolic disorders, or liver or kidney disease were excluded.

Plasma citrulline levels were measured across the three groups. In groups B and C, citrulline was measured once during routine controls (group B) or taking advantage of a requested blood test (group C). In group A, a second measurement was taken 3 to 6 months after starting the GFD as part of the longitudinal study. Citrulline levels before and after initiating GFD were compared, as were the levels at diagnosis with those from the other groups. Correlations were assessed between citrulline levels and clinical variables (age, anthropometric measurements, predominant clinical presentation at diagnosis in group A) as well as laboratory markers, including serological indicators.

Citrulline was measured in the post-absorptive state (after overnight fasting) using serum samples. Quantification was performed via ion-exchange chromatography with post-column ninhydrin reaction in a Biochrom30 automated analyzer.

The cutoff point for citrulline to discriminate CD was calculated using Youden’s index, with the gold standard for CD diagnosis being Marsh type 2 or 3 biopsy findings or, alternatively, tTG-IgA levels exceeding 10 times the upper limit of normal in groups A and C. Sensitivity (S), specificity (E), positive predictive value (PPV), negative predictive value (NPV), and receiver operating characteristic (ROC) curve analysis were determined.

The regression model for citrulline included the variables z-score for weight, height, and body mass index (BMI); Waterlow indices for weight and height; hemoglobin, hematocrit, alanine aminotransferase (ALT), and tTG-IgA. From the correlation matrix, the four variables with significance ≤ 0.2 were selected: hematocrit, ALT, tTG-IgA, and hemoglobin. Hemoglobin was subsequently excluded due to its relationship with hematocrit, which increased variance inflation factors and collinearity.

Differences in quantitative variables across the three groups were analyzed using ANOVA or the Kruskal–Wallis test, depending on normality and homoscedasticity assumptions. Correlations between numerical variables were evaluated using Pearson or Spearman coefficients, based on normality. Changes in citrulline levels before and after initiating the GFD in group A were analyzed with paired Student’s t-tests. A significance level of 0.05 was applied. Statistical analysis was performed using R software version 4.4.1 (The R Foundation, Vienna, Austria; https://cran.r-project.org) (accessed on 21 November 2024) with the OptimalCutpoints package [15].

Informed consent was obtained from the legal guardians of all participants. The study was approved by the Ethics Committee of our hospital.

## 3. Results

### 3.1. Description of the Main Characteristics of Patients Included in the Sample

**Figure 1:**
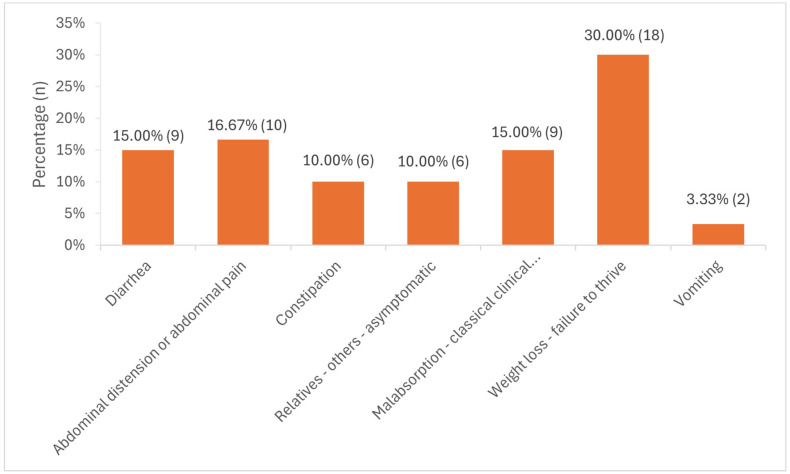
Predominant symptom at the time of celiac disease diagnosis in patients from groups A and B. The graph shows the percentage of patients, with the absolute number (n) indicated in parentheses.

The study included 93 patients distributed in three groups. Group A: 28 newly diagnosed celiac patients (30.1%). Although the planned sample size for the design was 30 patients per group. With the multicenter data collection, 28 patients were recruited in Group A. Of these 28 patients, 25 completed the follow-up. A second citrulline sample was not available for three of them. Group B: 32 celiac patients on a GFD for more than six months (34.4%). Group C: 33 healthy controls (35.5%). The mean age was 8.58 years, with 64.5% being female. Group A participants were significantly younger than those in Group C (*p* = 0.001) and Group B (*p* = 0.038). Table 1 summarizes the clinical and laboratory data of the groups. The most common symptom at diagnosis was failure to thrive (30%) (Figure 1), and 92.7% had DQ2-positive genetics.

### 3.2. Biopsies

Out of 60 celiac patients, biopsies were performed on 15 of them. All presented Marsh type 3 lesions. In two cases, pathology reports were unavailable, but biopsy records indicated compatibility with CD. Within Group A, biopsies were performed on 6 of 28 patients. Citrulline levels were lower in patients without biopsies (25.74 vs. 29.53 µmol/L), although the difference was not statistically significant (*p* = 0.41).

### 3.3. Citrulline Levels

Initial citrulline values ranged from 6.86 to 53 µmol/L, with a mean of 30.85 µmol/L. Group A patients had lower citrulline levels than those in Group B (27.13 vs. 32.42 µmol/L; *p* > 0.05). Citrulline levels were nearly identical between Group B and Group C (32.42 vs. 32.48 µmol/L; *p* > 0.05). No difference in citrulline levels was found between Group B and C, even when analyzing the six Group B patients with persistent positive tTG-IgA levels > 7 U/mL (*p* = 0.839).

Children under two years of age (n = 6), five of whom were in Group A and one in Group C, had significantly lower citrulline levels than older participants (19.48 vs. 31.63 µmol/L; *p* < 0.001). Within Group A, children under two years old had significantly lower citrulline levels than older members of the group (17.26 vs. 29.28 µmol/L; *p* < 0.001).

**Figure 2:**
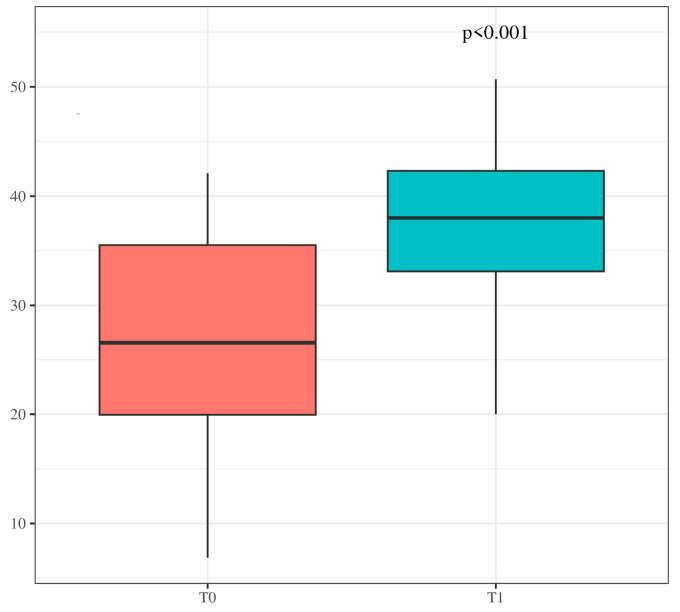
Box Plot. Increase in citrulline levels after a gluten-free diet in patients from Group A. T0: Baseline levels. T1: Levels after 3–6 months on a gluten-free diet. The lower edge of the box represents the first quartile (Q1), the value below which 25% of the data falls. The upper edge of the box represents the third quartile (Q3), the value below which 75% of the data falls. The thick line inside the box represents the median. The whiskers extend from the edges of the box (Q1 and Q3) to the maximum and minimum values.

The initiation of a GFD in Group A resulted in a significant increase in citrulline levels (from 27.13 to 37.43 µmol/L; *p* < 0.001) (Figure 2).

### 3.4. Citrulline Correlations

No correlation was found between baseline citrulline levels and somatometric data. However, the second measurement revealed a negative correlation between citrulline and weight. Table 2 presents statistically significant correlations. Among Group A patients, no relationship was observed between clinical presentation and citrulline levels at diagnosis (*p* = 0.807).

### 3.5. Cut-Off Point

A cutoff of 20 µmol/L was determined as indicative of disease. This yielded a sensitivity of 34.62%, specificity of 100%, positive predictive value of 100%, negative predictive value of 67.31%, and an area under the ROC curve of 0.645 (95% CI: 0.5–0.8).

In 25 of 28 Group A patients, follow-up citrulline measurements were conducted 3–6 months after GFD initiation, with all values exceeding 20 µmol/L.

### 3.6. Multivariate Analysis

A regression model for citrulline included hematocrit, ALT, and tTG-IgA as predictors. Only tTG-IgA reached statistical significance (*p* = 0.0033), with a negative coefficient. Each 1 U/mL increase in tTG-IgA corresponded to a mean decrease of 0.046 µmol/L in citrulline levels.

## 4. Discussion

The findings of our study indicate that citrulline levels at diagnosis in celiac patients are indicative of mucosal atrophy and increase significantly after initiating a GFD. This suggests that citrulline determination could be useful both at diagnosis and during follow-up, serving as a marker for mucosal recovery and adherence to treatment after the introduction of a GFD.

Normal plasma citrulline levels in adults range from 30 to 50 µmol/L. In children, levels are slightly lower due to reduced intestinal length [16], as corroborated by our findings. Various extraintestinal conditions can influence citrulline levels. For instance, renal insufficiency impairs the conversion of citrulline to arginine in the kidneys, resulting in elevated plasma concentrations. Conversely, citrulline production is reduced in septic patients [17,18].

Over the past two decades, several studies have demonstrated that reduced small intestinal length leads to decreased serum citrulline levels, including in cases of acute rejection following intestinal transplantation [19], short bowel syndrome [14,20,21], radiation-induced intestinal damage, and necrotizing enterocolitis in preterm neonates [22].

In 2003, Creen and colleagues were the first to report serum citrulline levels in adults with celiac disease and other causes of villous atrophy [23]. They identified a significant correlation between the extent and severity of atrophy and citrulline levels. Citrulline levels were observed to increase after one year of GFD, paralleling the normalization of serological markers. Moreover, citrullinemia correlated with albuminemia, calcium levels, and hemoglobin, but not with BMI or other laboratory parameters.

Our study additionally found correlations with iron levels and hematocrit, aligning with the improvement in iron absorption following mucosal recovery in the duodenum and jejunum. We also identified a previously undescribed negative correlation between citrulline and liver enzymes. This finding likely reflects the hypertransaminasemia frequently observed in celiac disease [4,24], which is typically due to nonspecific reactive hepatitis that resolves with GFD. Furthermore, in Group A, an inverse correlation was found between citrulline and z-score weight after 3–6 months of GFD, possibly related to initial challenges in adapting to the diet.

The most widely accepted cutoff value for citrulline indicative of disease appears to be 20 µmol/L [16], consistent with our results. Regarding the timing of citrulline normalization, our study observed that levels normalized within the first 3–6 months of GFD.

In our sample, it was not possible to evaluate the relationship between the degree of intestinal atrophy and citrulline levels in Group A patients, as all biopsied individuals were classified as Marsh type 3.

Miceli and collaborators, in a study on adults [25], found no significant differences in citrulline levels when comparing different degrees of intestinal damage or clinical presentations. They suggested this might be linked to the extent rather than the severity of the damage. Similarly, other authors have not observed correlations between citrulline levels and the degree of villous atrophy [26,27].

In 2008, Peters and colleagues [28] utilized a citrulline generation test (a solution of alanine-glutamine amino acids) that differentiated between healthy individuals and patients with varying degrees of villous atrophy. They also demonstrated the intestinal adaptation occurring over time in patients with short bowel syndrome. However, the study included a limited number of participants and it is not easily reproducible in pediatric clinical practice due to the need for numerous blood extractions.

The study by Bernini and colleagues [29] found that adult patients with potential celiac disease (positive serology for celiac disease but no histological lesions on biopsy) had significantly lower citrulline levels compared to healthy controls, with levels similar to those of patients with overt celiac disease. The authors suggested that potential celiac patients might already have undergone metabolic changes in the intestinal mucosa preceding atrophy, which could potentially justify earlier implementation of a GFD. In cases where a patient presents positive autoantibodies but a normal biopsy, this could either indicate patchy intestinal damage not captured during the biopsy or represent a potential celiac disease diagnosis.

In our study, the definition of patients in group B was based on GFD time rather than serological negativization. Significant differences were observed in tTG-IgA levels between groups. However, the absence of differences in citrulline levels between groups B and C remains consistent when selecting the six patients from group B whose tTG-IgA levels are still >7 U/mL (positive).

A pediatric study observed that citrulline levels in healthy controls were significantly higher than those in patients with mucosal atrophy, yet similar to those in celiac patients undergoing GFD treatment. Citrulline values of ≤27 µmol/L distinguished patients with mucosal atrophy from those without, with a sensitivity of 43% and a specificity of 90%. Moreover, it was more sensitive in patients under two years of age, who exhibited significantly lower citrulline levels. The study concluded that in selected patients with mucosal atrophy, citrulline was as reliable as tTG-IgA exceeding ten times the upper limit of normal [30]. This study followed the 2005 guidelines of the North American Society for Pediatric Gastroenterology, Hepatology, and Nutrition [11], with intestinal biopsies performed on all celiac patients. They identified a correlation between citrulline levels and the degree of atrophy, with 53 out of 63 biopsied patients showing Marsh 3 lesions. The study did not specify Marsh subtypes, leaving uncertainty about whether the differences observed were due to more severe degrees of atrophy. The reported cutoff of 27 µmol/L to detect atrophy was higher than that identified in our study. Consistent with our findings, patients under two years of age had significantly lower citrulline levels, possibly related to shorter intestinal length and greater intestinal damage.

In 2011, Ioannou and colleagues [31] published the first study analyzing the evolution of plasma citrulline levels in parallel with serology after the initiation of a GFD. Citrulline levels were lower in untreated celiac patients compared to those treated with a GFD, patients with intestinal symptoms but normal biopsy results, and healthy controls. However, the authors acknowledged an overlap in citrulline values between untreated patients and healthy controls. They found a correlation between citrulline levels and the severity of intestinal atrophy. Nonetheless, they also reported that citrulline levels in Marsh 3a patients were similar to those of healthy controls.

The study observed significant mucosal recovery at one month and normal citrulline levels by three months, at which point no differences were found compared to healthy controls. The authors suggested that once mucosal recovery reaches a certain threshold, it no longer correlates with plasma citrulline levels. Similarly to our study, they observed a significant increase in citrulline levels with GFD, indicating mucosal recovery within 3–6 months. Unlike adults, children achieve faster and more complete mucosal recovery, which could also be attributed to a lower initial degree of atrophy.

They found a stronger correlation between increased citrulline levels and a reduction in IgA AGA antibodies. However, this differentiation among various autoantibodies was not observed in our study.

In another pediatric study, plasma levels of citrulline and arginine were correlated with the severity of intestinal mucosal damage in celiac patients. The study concluded that fasting plasma citrulline measurement is a good marker for enterocyte mass reduction, identifying a cutoff of 20 µmol/L with a sensitivity of 72%, specificity of 76%, positive predictive value (PPV) of 73%, and negative predictive value (NPV) of 75% (false-positive rate: 27%; false-negative rate: 25%) [32]. In our study, the same cutoff value of 20 µmol/L was identified. However, the sensitivity was lower (34.62%), while specificity was maximal (100%), with a PPV of 100% and a comparable NPV (67.31%). Practically, these results indicate that while the test does not produce false positives (specificity = 100% and PPV = 100%), its ability to detect all patients with celiac disease is limited (sensitivity = 34.62%). The area under the ROC curve (0.645) reflects a modest but statistically significant discriminatory capacity to differentiate between controls and newly diagnosed celiac patients.

Although the cutoff value calculated using the Youden index is 20, it is notable that sensitivity is low. Many patients in Group A already present values above 20 in the initial measurement.

Consistent with these results, Rahmani and collaborators published a study including 118 children diagnosed with CD who were referred for biopsy. They found that higher Marsh classifications were associated with decreased citrulline levels and increased tTG-IgA levels [33]. However, this cohort might be biased, as it consisted of already-diagnosed patients referred to a specialized center for biopsy, potentially representing cases with more severe intestinal atrophy.

Another study conducted in India concluded that plasma citrulline levels < 30 µmol/L could predict villous abnormalities and potentially obviate the need for duodenal biopsies in 78% of celiac patients [34].

## 5. Conclusions

The strengths of this study include its multicenter and prospective design. However, there are some limitations. Plasma citrulline levels could not be correlated with histopathological findings or the stage of the lesion (Marsh classification) in all patients. According to the 2012 ESPGHAN criteria, not all patients require biopsy for CD diagnosis. These criteria were applied rigorously in the present study. Consequently, the group of celiac patients included individuals with varying degrees of mucosal damage. If mild intestinal atrophy was overrepresented in this group, citrulline levels might have been only slightly reduced, potentially affecting the citrulline values observed.

Additionally, Group B patients were selected based on the duration of gluten exclusion, not serological values. Thus, some patients with residual mucosal atrophy and decreased citrulline levels might have been included.

In conclusion, plasma citrulline determination appears to be a promising marker for mucosal recovery in the follow-up of diagnosed celiac patients adhering to a gluten-free diet.

## Figures and Tables

**Table 1:** Clinical and Analytical Data Across the Different Groups.

	Group A	Group B	Group C	*p*
n	28	32	33	
Age in years (St. dev)	6.35 (3.85)	8.9 (4.04)	10.15 (3.95)	*
Women (%)	20 (71.42%)	24 (75.00%)	16 (48.48%)	0.054
Weight Z-score (St. dev)	−0.62 (1.28)	−0.22 (1.00)	−0.18 (1.19)	0.121
Height Z-score (St. dev)	−0.95 (1.48)	−0.70 (1.40)	−1.09 (1.34)	0.236
BMI Z-score (St. dev)	−0.25 (1.22)	−0.01 (0.79)	0.29 (1.03)	0.101
Waterlow weight (St. dev)	95.28 (13.06)	99.64 (9.19)	109.35 (17.63)	0.01 ++
Waterlow height (St. dev)	96.59 (6.45)	97.12 (5.79)	95.09 (5.81)	0.234
IgA g/L (St. dev)	1.48 (1.16)	0.90 (0.58)	0.98 (0.67)	0.345
DGP-IgG U/mL (St. dev)	124.68 (101.39)	23.33 (72.33)	2.81 (4.80)	<0.001 +/++
tTG-IgA U/mL (St. dev)	128.98 (48.24)	6.11 (9.23)	0.35 (0.25)	<0.001 +/++/**
Citrulline micromoles/L (St. dev)	27.13 (10.07)	32.42 (5.67)	32.48 (7.84)	0.11
Hemoglobin g/dL (St. dev)	12.22 (1.32)	13.47 (1.06)	13.39 (1.44)	0.001 +/++
Hematocrit % (St. dev)	36.65 (3.4)	38.85 (2.72)	38.97 (3.87)	0.026 ++
AST U/L (St. dev)	34.33 (21.61)	26.89 (12.25)	25.19 (7.63)	0.428
ALT U/L (St. dev)	29.76 (17.68)	20.56 (16.78)	17.03 (6.99)	0.001 +/++
Iron mg/L (St. dev)	66.32 (38.75)	93.81 (29.98)	80.20 (30.78)	0.022+
Ferritin ng/mL (St. dev)	22.87 (18.93)	45.44 (19.44)	47.70 (25.17)	<0.001 +/++
Albumin g/L (St. dev)	43.85 (3.89)	46.75 (1.96)	47.21 (3.22)	0.009 ++
TSH mU/mL (St. dev)	2.76 (1.90)	3.12 (1.51)	3.03 (2.00)	0.302

Group A: patients with a recent diagnosis of celiac disease. Group B: celiac patients on a gluten-free diet for more than 6 months. Group C: controls. *p*: *p*-value of the differences between groups. The table presents data as mean (standard deviation). St dev: Standard deviation. BMI: body mass index. IgA: immunoglobulin A. DGP-IgG: anti-deamidated gliadin antibodies IgG. tTG-IgA: anti-tissue transglutaminase IgA antibodies. AST: aspartate aminotransferase. ALT: alanine aminotransferase. TSH: thyroid-stimulating hormone. * The groups exhibit different behaviors, as discussed in the text. For parameters where the groups behave differently group A has been used as the reference. When a difference in a parameter is found between groups A and B, it is marked with a cross (+). If differences are identified between groups A and C, two crosses are used (++). For parameters where differences exist between groups B and C, two asterisks are used (**).

**Table 2:** Correlation of Citrulline with Somatometric and Analytical data.

Variables	Correlation Coefficient	*p*-Value
Citrulline and Age	0.260	0.012
Citrulline2 and Weight Z-score2	−0.569	0.005
Citrulline and Hemoglobin	0.235	0.034
Citrulline and Hematocrit	0.250	0.024
Citrulline and Iron	0.333	0.012
Citrulline and AST	−0.372	0.003
Citrulline and ALT	−0.350	0.002
Citrulline and IgA	−0.399	0.021
Citrulline and DGP	−0.349	0.006
Citrulline and tTG-IgA	−0.273	0.018

Abbreviations. Citrulline2: citrulline (μmol/L) after 3–6 months on a gluten-free diet; AST: aspartate aminotransferase (U/L); ALT: alanine aminotransferase (U/L); IgA: immunoglobulin A (g/L); DGP: anti-deamidated gliadin antibodies (U/mL); tTG-IgA: anti-tissue transglutaminase antibodies (U/mL).

## Data Availability

The original contributions presented in this study are included in the article. Further inquiries can be directed to the corresponding author.
